# *Spirulina* and *Chlorella* Dietary Supplements—Are They a Source Solely of Valuable Nutrients?

**DOI:** 10.3390/ijms262110468

**Published:** 2025-10-28

**Authors:** Małgorzata Sochacka, Bartosz Kózka, Eliza Kurek, Joanna Giebułtowicz

**Affiliations:** 1 Department of Drug Chemistry, Pharmaceutical and Biomedical Analysis, Faculty of Pharmacy, Medical University of Warsaw, Banacha 1, 02-097 Warsaw, Poland; malgorzata.bogucka@wum.edu.pl (M.S.); bartosz.kozka@wum.edu.pl (B.K.); 2 Centre of Biological and Chemical Sciences, Faculty of Chemistry, University of Warsaw, Żwirki i Wigury 101, 02-089 Warsaw, Poland

**Keywords:** contaminants, dietary supplements, environment, heavy metals, mass spectrometry, pharmaceuticals

## Abstract

*Spirulina* and *Chlorella* are nutrient-rich microalgae widely consumed as dietary supplements; however, their high biosorption capacity raises concerns regarding the accumulation of environmental contaminants. This study analyzed 52 commercially available *Spirulina* and *Chlorella* products (29 conventional, 23 organic) to assess the co-occurrence of heavy metals and pharmaceutical residues, as these two classes of contaminants represent distinct yet complementary indicators of environmental pollution—heavy metals reflect long-term inputs from natural and industrial sources, while pharmaceuticals signal more recent contamination linked to human activity and wastewater discharge. To the best of our knowledge, this is the first study to investigate the presence of pharmaceutical residues—including cardiovascular drugs, antidepressants, antibiotics, and sulfonamides—in both conventional and organic formulations of microalgae-based dietary supplements. The analyses were performed using Inductively Coupled Plasma Mass Spectrometry and liquid chromatography coupled to tandem mass spectrometry. Aluminum, manganese, strontium, and zinc were the dominant trace elements. All samples complied with EU regulatory limits for toxic metals. More importantly, a wide range of pharmaceutical residues was detected in the supplements. Caffeine was the most frequently found compound, followed by metronidazole, carbamazepine, benzocaine, and tramadol. Particular concern is raised by the calculated TWI (% of tolerable weekly intake) for aluminum. Principal Component Analysis revealed significant compositional differences between *Spirulina* and *Chlorella* products, with vanadium notably elevated in conventionally cultivated *Spirulina*. Surprisingly, no significant differences were observed between organic and conventional products within each algal type. Our findings provide a novel contribution to the field by highlighting the presence of pharmaceutical residues in microalgae-based supplements and addressing a critical knowledge gap concerning potential chronic exposure to these contaminants through dietary intake.

## 1. Introduction

Dietary supplements are currently among the fastest-growing products in the global market, providing a convenient way to enhance the daily intake of essential nutrients, such as vitamins and minerals [1]. However, unlike pharmaceutical products, dietary supplements are not subject to the same rigorous quality control standards, which can make them susceptible to contamination with harmful substances, including pharmaceuticals and heavy metals such as cadmium, lead, and mercury [2,3]. This lack of stringent oversight raises significant safety concerns and underscores the need for enhanced regulatory scrutiny and quality assurance in the production of dietary supplements [3].

Supplements derived from microalgae or cyanobacteria, particularly *Spirulina* and *Chlorella*, have gained significant popularity in European markets due to their impressive nutritional profiles. *Spirulina platensis*, cyanobacteria, is one of the earliest life forms on Earth, dating back over 3.6 billion years. It is renowned for its high digestibility and rich nutrient content, which includes essential amino acids, proteins, vitamins (such as beta-carotene, carotenoids, vitamin B12, and other B vitamins), and vital minerals. As such, *Spirulina* has become a popular supplement for individuals seeking a natural and effective way to improve their nutritional intake [4]. Its diverse nutrient profile provides significant health benefits, including strong antioxidant, antiviral, and cytostatic properties. Regular supplementation with *Spirulina* has been shown to lower blood pressure, reduce blood lipid and glucose levels, and support weight loss [5,6]. Additionally, it promotes the growth of beneficial gut microbiota, essential for digestive health, and offers protective effects for the kidneys against damage from heavy metals and certain medications [7,8].

*Chlorella vulgaris*, microalga, is widely recognized for its health benefits and has been consumed traditionally in many East Asian countries. Recently, it has attracted attention for its potential to enhance the nutritional content of conventional foods. Research highlights its antiviral properties, including anti-SARS-CoV-2 activity, as well as antibacterial and anti-tumor effects [9,10,11]. *Chlorella* has also been shown to reduce the risk of chronic diseases such as hypertension, diabetes, and cardiovascular conditions, while serving as a preventive agent against macular degeneration, cataracts, anemia, and gastric ulcers [12,13].

While microalgae supplementation offers numerous health benefits, it is essential to consider the potential risks associated with contamination. *Spirulina* and *Chlorella* are able to bioaccumulate heavy metals, such as lead and cadmium, at concentrations significantly higher than those found in their environment [14,15]. This bioaccumulation occurs rapidly and can pose serious health risks if contaminated algae are consumed. Therefore, it is crucial to monitor and control the levels of these metals in *Spirulina* and *Chlorella* products to ensure their safety for human consumption [16]. Heavy metals ingested through food can lead to allergic reactions and toxicity, affecting nearly every organ system. These metals interfere with protein synthesis and ATP production, causing damage to cell membranes and organelles, including mitochondria, lysosomes, and the nucleus. This cellular damage may result in toxicity affecting the digestive, hematopoietic, cardiovascular, respiratory, and nervous systems, and some heavy metals are known carcinogens [17].

While environmental pollution, including heavy metal contamination, has been extensively studied, the presence of pharmaceutical residues in food products derived from aquatic sources remains a relatively underexplored issue [18]. Pharmaceuticals have only recently emerged as contaminants of growing concern. The rapid expansion of the global pharmaceutical industry—valued at approximately 1.2 trillion USD in 2018, a significant increase from 390 billion USD in 2001—has raised alarm regarding the impact of pharmaceutical compounds on ecosystems and food safety. In Poland, the pharmaceutical market grew at an average annual rate of 4.8% between 2016 and 2021, highlighting the increasing consumption of pharmaceuticals and the potential environmental consequences [19].

The widespread use of pharmaceuticals in both human and veterinary medicine has led to their accumulation in the natural environment, particularly in aquatic systems [20]. One of the primary pathways through which pharmaceutical contaminants enter water bodies is municipal wastewater, which contains active pharmaceutical compounds and their metabolites. These substances reach wastewater treatment plants mainly through human excretion and the improper disposal of unused or expired medications [21]. Due to the physicochemical properties of many pharmaceuticals—such as solubility in water, low sorption potential, and resistance to biodegradation—conventional water treatment processes are often ineffective in completely removing them. Additionally, pharmaceutical production facilities contribute to environmental pollution by releasing contaminants during manufacturing processes [22,23].

Although awareness of pharmaceutical contamination in aquatic systems is increasing, limited information is available regarding the accumulation of pharmaceutical residues in dietary supplements derived from microalgae and cyanobacteria, such as *Spirulina* and *Chlorella*. These algae, often cultivated in open water systems, can be vulnerable to absorbing contaminants from their surroundings. Recent studies have shown that aquatic plants, such as *Lemna minor*, accumulate pharmaceuticals and thus participate in the removal of these compounds from water, demonstrating their potential role in natural and engineered wastewater treatment processes [24]. However, the capacity of *Spirulina* and *Chlorella* to absorb pharmaceuticals remains poorly understood, making it difficult to estimate the concentrations of these contaminants that may be present in algal-based dietary supplements. Moreover, the potential health risks associated with chronic exposure to pharmaceutical residues through such supplements remain largely unexplored. Toxicity levels may vary depending on exposure routes, concentrations, and frequency of intake, with individual factors such as age and health status influencing susceptibility to these contaminants. Given these uncertainties, systematic monitoring of pharmaceutical residues in dietary supplements and their potential impact on human health is a crucial process [25].

To the best of our knowledge, no data are currently available on the presence of pharmaceutical residues in microalgae-based dietary supplements. Moreover, although heavy metals such as lead, arsenic, and cadmium have been detected in such products worldwide, information specific to supplements available on the Polish market remains limited. Given the growing popularity of these supplements and their reputation as natural health products, we decided to include them in our analysis. This study addresses this gap by evaluating the presence of various pharmaceuticals—including cardiovascular drugs, antidepressants, antibiotics, and sulfonamides—alongside trace elements and heavy metals (Al, Ba, Cd, Co, Cr, Cu, Ga, Mo, Ni, Pb, Rb, Sr, Tl, V, Zn) in both conventional and organic formulations of *Spirulina* and *Chlorella* supplements available in Poland.

## 2. Results

### 2.1. Selected Trace Elements and Heavy Metals Determination

The concentrations of the detected heavy metals and trace elements in the samples are provided in Table A1. Elements that were below the detection limit in all tested samples (Ag, Be, Bi, In, Li) were excluded from further analysis.

The most abundant metals detected in dietary supplements containing *Chlorella* were Al, Mn, Sr and Zn. The mean concentrations of Al, Mn, Sr and Zn were 148 ± 163 µg/g; 75 ± 31 µg/g; 16 ± 16 µg/g and 18.9 ± 8.2 µg/g, respectively. The highest Al concentration was observed in sample no 18 (Taiwan origin), while the highest levels of Mn and Zn were found in samples no 22 (Chinese origin), no 3 (Poland origin) and no 20 (Denmark origin), respectively. The least abundant metal was Tl with the highest level of 0.015 ± 0.015 µg/g. The high standard deviations resulted from substantial variations in mineral content observed across individual samples.

In the analyzed dietary supplements containing *Spirulina*, similar to those with *Chlorella*, the predominant elements were Al, Mn, Sr, Zn. Their mean concentrations were: 269 ± 267 µg/g; 47 ± 37 µg/g; 18.0 ± 9.8 µg/g and 18.1 ± 8.7 µg/g, respectively. The highest Al concentration was detected in sample no 39 (China origin), while the highest Mn, Sr and Zn levels were observed in samples no 52 (USA origin), no 36 (Poland origin) and no 30 (China origin), respectively. The least abundant metal was Cd with the highest concentration of 0.059 ± 0.081 µg/g.

Table A2 and Table A3 show the content of the studied minerals depending on the method of cultivation of *Chlorella* and *Spirulina.* In the case of all elements except V in *Spirulina* samples, the differences between the conventional and organic growing groups were not statistically significant (*p* > 0.05). *Spirulina* supplements with conventional growing algae had a significantly higher content of V compared to the organic group of supplements (*p* = 0.023271).

Table 1 presents the estimated weekly intake of trace elements and toxic metals (expressed in µg/week and mg/week), along with their percentage contribution to the maximum permissible intake (PTWI) levels for each detected element (Table A4). It can be observed that for the recommended daily dose, these preparations contribute to a high intake of Al, Mn, Zn, and Cr, indicating that their consumption may significantly approach the tolerable weekly intake (TWI) of these elements [26,27,28,29,30,31,32,33]. In the case of Al content, the consumption of powdered *Chlorella* preparations of supplement no 2 (Poland origin), no 16 (Chinese origin) and 23 (Chinese origin) provide a contribution percentage of 25.04%, 28.92%, 17.13% of the TWI of Al (2.8 μg/kg bw/day) [26]. The same consumption of *Spirulina* preparations of the no 39 (Chinese origin), no 49 (Chinese origin) and no 52 (USA origin) supplements represents a percentage of contribution of 56.10%, 22.57%, and 22.01%, respectively, of the TWI of Al.

### 2.2. Pharmaceutical Determination

**Figure 1:**
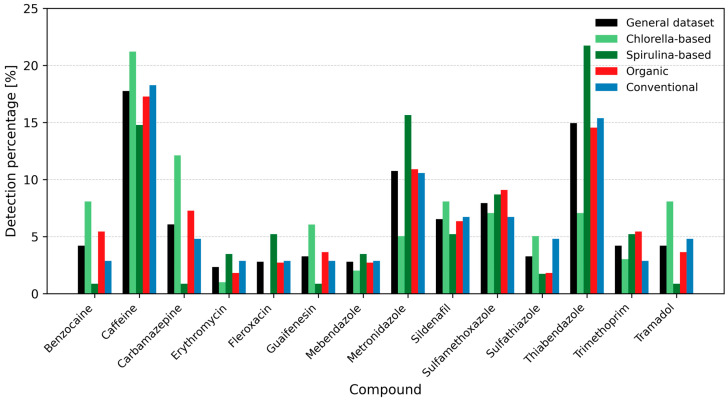
Detection percentage (Det.%) of selected pharmaceuticals in *Chlorella*- and *Spirulina*-based dietary supplements. Bars represent detection frequencies in the general dataset and separately for *Chlorella*, *Spirulina*, organic- and conventional products (overall detection > 2%). Color coding: black—general dataset; light green—*Chlorella*; dark green—*Spirulina*; red—organic; blue—conventional. Detection percentage was calculated as (number of detections/total detections) × 100.

As shown in Figure 1, caffeine was the most frequently detected pharmaceutical across all supplement categories, with detection rates exceeding 20% in *Chlorella*-based products, under 15% in *Spirulina*-based products, and over 15% in general and conventional supplements. Carbamazepine was also commonly found, with detection frequencies ranging from approximately 2% to >5% across almost all groups. Thiabendazole showed a notably high occurrence in *Spirulina*-based supplements (>20%), while metronidazole was present in over 15% of *Spirulina* samples. Other pharmaceuticals, including sildenafil and sulfamethoxazole, appeared at lower frequencies. Detection patterns varied by supplement type, with higher rates of caffeine, carbamazepine, and benzocaine observed in *Chlorella*-based products, and thiabendazole and metronidazole predominating in *Spirulina*-based supplements.

Table 2 presents summary statistics for detected pharmaceuticals. The highest maximum concentrations and widest ranges were recorded for sulfathiazole, benzocaine, and caffeine. Mean concentrations often exceeded medians, indicating skewed distributions and the presence of samples with exceptionally high residue levels. Overall, the data reveal considerable variability in pharmaceutical concentrations across supplements. Summary statistics for the detected pharmaceuticals, distinguishing between sample types (*Chlorella* or *Spirulina*), are presented in Table A5.

**Figure 2:**
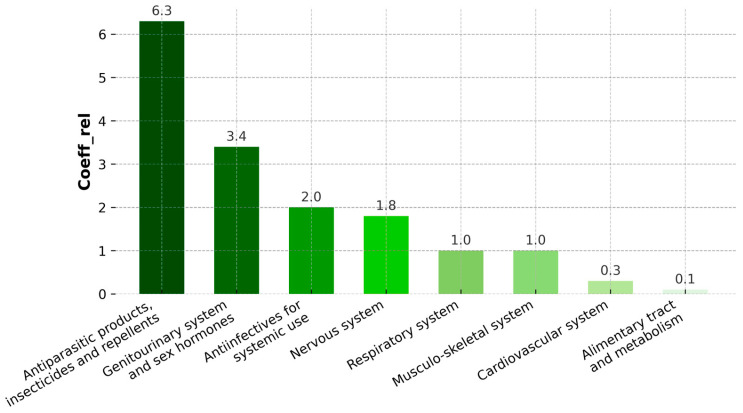
Distribution of pharmaceutical groups detected in *Spirulina* and *Chlorella* dietary supplements according to the first level of the ATC Classification System. The relative frequency coefficient (coeff_rel_) indicates the frequency of occurrence of each pharmaceutical group. The values of relative coefficients for “systems” groups.

The distribution of pharmaceutical groups (ATC) detected in *Spirulina* and *Chlorella* dietary supplements is presented in Figure 2. The highest relative frequency coefficient (coeff_rel_ > 6) was observed for antiparasitic products, insecticides, and repellents, primarily represented by thiabendazole and mebendazole. This group was followed by compounds related to the genitourinary system and sex hormones, with sildenafil as the most frequently detected. Moderate detection frequencies were noted for anti-infectives for systemic use and nervous-system agents. Lower frequencies were recorded for compounds linked to the respiratory, musculoskeletal, cardiovascular, and alimentary systems.

**Figure 3:**
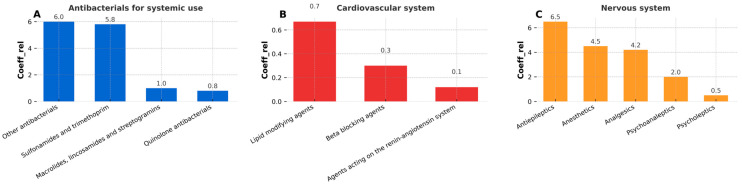
Relative detection frequency coefficient for three most important groups: (**A**) antibacterials for systemic use, (**B**) cardiovascular system, (**C**) nervous system.

Figure 3 focuses on substances targeting the anti-infective, cardiovascular, and nervous systems, as these therapeutic groups are among the most commonly used in both human and veterinary medicine. Notably, these classes also showed the highest coefficient values in the study, indicating a greater prevalence or intensity of contamination. Among the anti-infective agents, only antibacterials were detected—no antivirals or antimycotics were found in any sample.

As shown in Figure 3A, tetracyclines, β-lactam antibiotics, and antituberculosis drugs were absent in all tested samples. Instead, the category labeled as “other antibacterials” dominated, which includes compounds such as furazidin, metronidazole, tiamulin, and vancomycin. Within this subgroup, metronidazole was the most frequently detected pharmaceutical. The group of sulfonamides and trimethoprim also showed a high relative detection coefficient (coeff_rel_), nearly equal to that of other antibacterials, indicating their widespread presence across samples

For cardiovascular system drugs (Figure 3B), the highest coeff_rel_ was recorded for lipid-modifying agents, represented solely by atorvastatin in this study. However, the absolute coeff_rel_ values for all cardiovascular drugs remained low, suggesting these compounds were detected only sporadically. Beta-blockers and agents acting on the renin–angiotensin system were detected even less frequently.

Overall, these data demonstrate that antibacterial agents are more prevalent in dietary supplements based on *Chlorella* and *Spirulina* than drugs from other therapeutic groups.

The analysis of pharmaceuticals associated with the nervous system (Figure 3C) revealed the most diverse composition among all evaluated therapeutic groups, as evidenced by the identification of five distinct subgroups. The highest coeff_rel_ within this category was observed for antiepileptic drugs, with carbamazepine emerging as the predominant compound. Its frequent detection is consistent with its widespread usage and environmental persistence. The second-highest coeff_rel_ values were recorded for anesthetics and analgesics, represented, respectively, by benzocaine and tramadol—both well-known and widely used pharmaceuticals. Psychoanaleptics ranked third in terms of coeff_rel_, primarily due to the detection of caffeine. Despite being the most frequently detected individual compound across all samples (based on detection percentage), the overall coeff_rel_ for psychoanaleptics remained moderate. This paradox is attributed to the broad heterogeneity of substances classified under this group and the low incidence of detection for other representatives beyond caffeine. Finally, psycholeptics displayed the lowest relative frequency among nervous-system pharmaceuticals, with only tiapride detected—and that in merely two samples. This suggests either lower environmental prevalence or more efficient degradation/removal mechanisms for this subclass.

### 2.3. Comprehensive Assessment of Pharmaceuticals and Metals of Algae-Based Samples

#### 2.3.1. Principal Component Analysis

**Figure 4:**
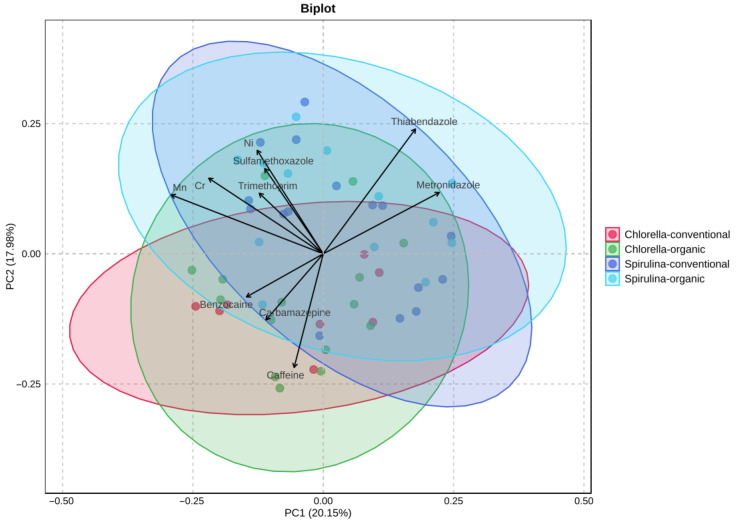
Principal Component Analysis (PCA) of *Chlorella* and *Spirulina* samples based on selected pharmaceutical residues and trace metal content.

PCA was applied to assess the variation in pharmaceutical residues and trace metal content among four supplement groups: *Chlorella*-organic, *Chlorella*-conventional, *Spirulina*-organic, and *Spirulina*-conventional (Figure 4). The first two principal components explained 38.13% of the total variance (PC1: 20.15%; PC2: 17.98%). No differences were observed between products derived from organic and conventional cultivation. Although visual clustering was limited, partial group separation was observed with *Spirulina* and *Chlorella* samples—especially conventional. The variables most strongly contributing to differentiation were thiabendazole, metronidazole, carbamazepine, benzocaine, and caffeine.

The relatively low variance explained by PC1 and PC2 reflects the large number of variables and the chemical diversity of the analyzed samples. The goal of PCA was to obtain combinations of components that provide meaningful sample separation rather than to maximize total explained variance.

#### 2.3.2. Hierarchical Clustering

**Figure 5:**
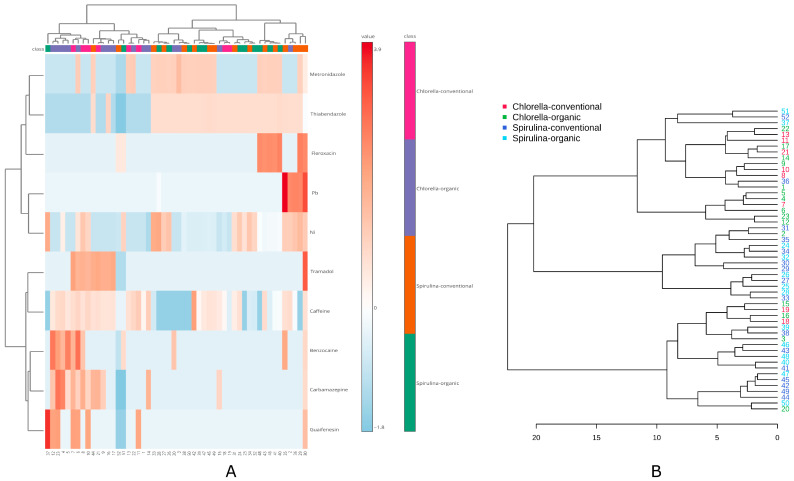
Clustering of factors and samples: (**A**)—Heatmap, (**B**)—Dendrogram with the samples clustering.

To further explore and validate the relationships identified in previous analyses, hierarchical clustering was applied. This unsupervised method reveals natural groupings among samples or variables based on similarity, thereby enhancing the interpretation of observed patterns. The hierarchical clustering analysis combined with heatmap visualization (Figure 5A) revealed a diversified distribution of pharmaceutical residues and metal content across the studied algal supplements. Although overall grouping was relatively weak, a discernible separation between *Spirulina* and *Chlorella* samples could be observed. Samples classified as *Spirulina* (both organic and conventional) were characterized by higher concentrations of Ni, Pb, fleroxacin, thiabendazole, and metronidazole, which contributed to their grouping in the heatmap. In contrast, *Chlorella* samples were more strongly associated with the presence of benzocaine, carbamazepine, and guaifenesin.

The corresponding dendrogram (Figure 5B) showed high branch complexity (strong furcation), indicating substantial variability between individual products. Despite this heterogeneity, the clustering structure allowed for the identification of intra-type similarities, with most *Spirulina* and *Chlorella* samples forming relatively distinct clusters. These results confirm the influence of both product type and contamination profile on the classification of algal food supplements.

#### 2.3.3. Partial Least Squares Discriminant Analysis

**Figure 6:**
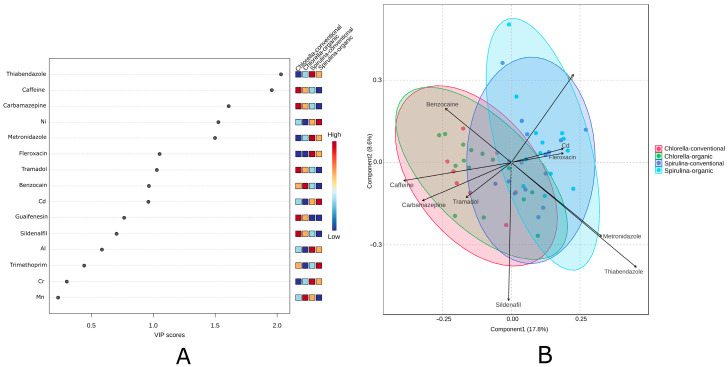
Diagram of PLS-DA analysis: (**A**) Variable Importance in Projection (VIP) scores; (**B**) PLS-DA biplot showing the separation of *Spirulina* and *Chlorella* supplements by microalgae type and cultivation method. Ellipses represent 95% confidence intervals. The PLS-DA model showed acceptable performance with R^2^ ≈ 0.55, Q^2^ ≈ 0.33.

PCA and hierarchical clustering were applied to explore the data structure in an unsupervised manner. To further investigate group differences, PLS-DA was conducted. PLS-DA is a supervised multivariate method that maximizes the separation between predefined classes while identifying variables most responsible for this differentiation. It is particularly useful when variables are collinear or when the number of predictors exceeds the number of observations. The Variable Importance in Projection (VIP) analysis from the PLS-DA model (Figure 6A) identified key compounds responsible for differentiating algal product samples. The most influential variables (VIP > 1) were primarily pharmaceuticals, with thiabendazole, caffeine, and carbamazepine showing the highest impact on sample classification. Nickel was the only metal among the top-ranking variables, suggesting its notable role despite the overall dominance of pharmaceutical residues. Organic *Spirulina* samples clustered separately from conventional *Chlorella* (Figure 5B). As shown in Figure 6A, the relationships and patterns are further visualized in Figure 6B. These results are consistent with the observations from the unsupervised PCA, confirming that some pharmaceutical and metal contents differ between *Spirulina* and *Chlorella* preparations.

#### 2.3.4. Correlation Analysis

**Figure 7:**
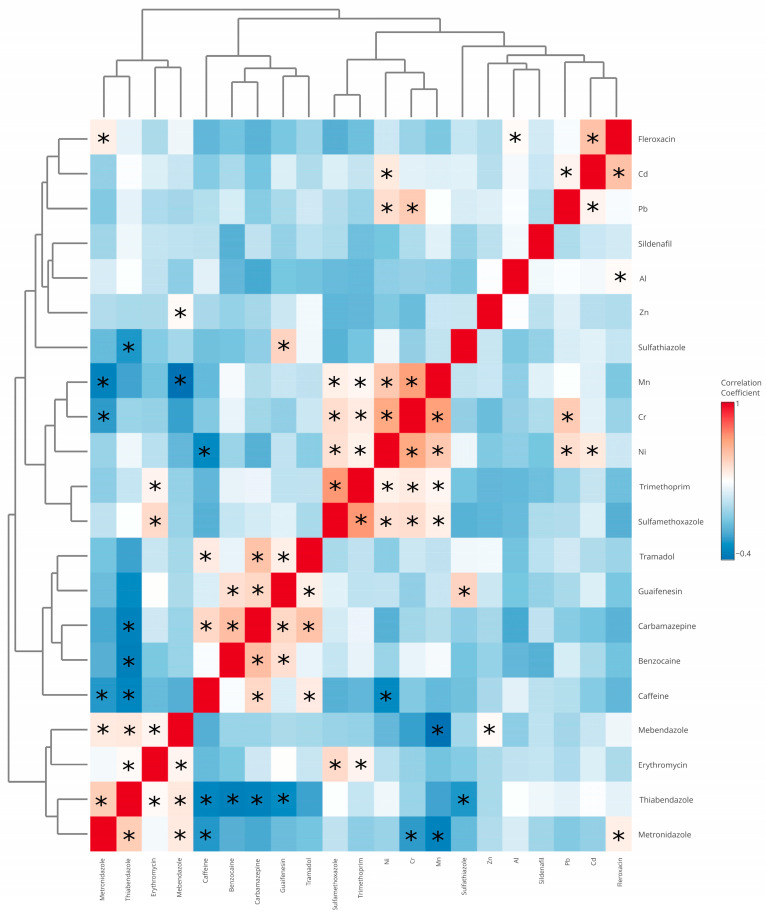
Heatmap of correlations. Asterisks indicate statistically significant correlations (*p* < 0.05).

Given the variable groupings revealed by hierarchical clustering, correlation analysis was performed to further investigate co-occurrence patterns and identify contaminants that tend to be associated with one another. The correlation analysis (Figure 7) was conducted to explore potential relationships between the detected pharmaceuticals and metal elements in the algal supplements. Overall, no strong (|r| > 0.7) or very strong (|r| > 0.9) correlations were observed. Most correlation coefficients indicated weak to moderate associations (|r| = 0.281–0.653), suggesting relatively independent occurrence of individual contaminants. The strongest observed correlation was between sulfamethoxazole and trimethoprim (r = 0.676), which is consistent with their common co-occurrence in pharmaceutical formulations. Notable correlations were also found between nickel and chromium (r = 0.626), as well as manganese and chromium (r = 0.653), potentially reflecting shared environmental or production-related contamination sources. These findings indicate that, although some compounds tend to co-occur, there is no clear pattern of systematic contamination, and the diversity of profiles further highlights the complexity of the analyzed products.

## 3. Discussion

In recent years, concerns over the contamination of dietary supplements, particularly algae-based products such as *Spirulina* and *Chlorella*, have grown due to their potential to accumulate heavy metals, pharmaceuticals, and trace elements from the environment. This study aims to evaluate the levels of such contaminants in these algae species, which are increasingly popular in the Polish dietary supplement market due to their purported health benefits. By examining the presence of 17 metals (including Pb and Cd) and several pharmaceutical residues in supplements of *Spirulina* and *Chlorella*, we aimed to characterize their contaminant profiles and explore potential implications for product quality and safety.

Among the pharmaceutical compounds detected, caffeine was the most frequently detected, present in all tested samples. This compound’s widespread occurrence reflects its pervasive use in both pharmaceuticals and everyday consumer products, such as beverages and over-the-counter medications. Consequently, caffeine is commonly found in municipal wastewater effluents and surface waters, where it can serve as a marker of contamination [34,35]. Its consistent presence in all algae samples suggests that algae may act as bioaccumulators of caffeine and other pharmaceuticals, thus serving as indicators of environmental pollution. Studies Vieira et al. and Dafouz et al. have similarly detected caffeine in various aquatic environments, underscoring its persistence in the ecosystem and highlighting its potential to accumulate in organisms at different trophic levels [34,36]. The widespread presence of caffeine in *Spirulina* and *Chlorella* is therefore not surprising, reflecting the ubiquity of this compound in water sources impacted by human activity [37,38].

The predominance of antiparasitic and anti-infective agents in the analyzed samples suggests potential environmental contamination, particularly from agricultural and aquaculture sources [39]. Among all therapeutic classes, antiparasitic compounds were detected most frequently, aligning with findings by Felis et al. [40], who reported their presence in water bodies influenced by runoff from livestock farming. The consistent detection of agents such as albendazole and mebendazole, previously identified near agricultural zones, indicates that these substances may persist in the environment and bioaccumulate in lower trophic organisms, including microalgae. Although these compounds were frequently detected, their concentrations remained low—well below levels considered pharmacologically active in humans. 

In addition, the antibiotic metronidazole was the most commonly detected antibacterial agent in our study. This result is consistent with previous reports by Mejías et al. and Giebułtowicz et al., who identified metronidazole and related antibiotics in surface waters and aquatic organisms, reinforcing the notion of diffuse environmental exposure pathways [41,42,43]. The detection of metronidazole in algae is particularly concerning, as this drug is now used more frequently in veterinary medicine than in human therapy. Its relatively rare application in human medicine, contrasted with its high prevalence in algal samples, suggests that animal-related sources may play a notable role in its release into aquatic environments. Although the concentrations found in our study were low and unlikely to cause direct therapeutic effects, their occurrence still raises questions about contamination pathways and underscores the need for stricter oversight of veterinary pharmaceutical use and waste management practices to prevent transfer into the food chain [44].

Within the cardiovascular category, atorvastatin, a lipid-modifying agent, was most frequently identified in our algae samples. The presence of atorvastatin is notable, as it has been detected in several studies of pharmaceutical contamination in water, including research by Kingsbury et al., who found statins in the effluents of wastewater treatment plants [45]. Statins are known to have relatively high persistence in the environment, and their accumulation in algae could have implications for environment. However, in our study, the concentrations of statins detected were very low, well below any levels that could pose a risk to human health or exert pharmacological effects [46]. In addition, our study revealed the presence of several neuroactive drugs, including carbamazepine, benzocaine, and tramadol. Carbamazepine, an anticonvulsant, was detected most frequently among these neuroactive compounds. These results are consistent with findings from Hejna et al., who reported the widespread presence of carbamazepine in the aquatic environment, largely due to its high environmental persistence and excretion by humans [47]. In algae-derived dietary supplements, tramadol and benzocaine—both pharmaceuticals commonly used in pain management—were also detected at low concentrations. These compounds had previously been reported in surface waters [48].

The detection of sulfathiazole, categorized as an anti-infective for systemic use (ATC classification), in one of the analyzed supplements at a relatively high concentration is noteworthy. Although its use in human medicine has declined, it remains prevalent in veterinary applications, which likely accounts for its occurrence in algae-derived products [49]. Although the concentration found was well below the minimal inhibitory concentration (MIC) equal to 250 µg/L and thus unlikely to exert therapeutic or bactericidal effects, it was close to levels considered sufficient for resistance selection [50]. According to Gullberg et al., resistant bacterial strains may be selected at concentrations as low as 1/100 to 1/230 of MIC [49]. In fact, the concentration observed in our sample was close to that threshold, suggesting that, although not therapeutically active or bactericidal, the compound may still promote the selection of resistant bacteria, particularly upon chronic low-dose exposure. Such findings highlight the need for stricter control and regular screening of dietary supplements for pharmaceutical contaminants.

While *Spirulina* and *Chlorella* are widely considered beneficial for human health, our results suggest that the contamination of these products with pharmaceuticals, including antibiotics, antiparasitics, cardiovascular drugs, and neuroactive substances, warrants increased scrutiny [51]. Comparing our results to the existing literature, it is evident that pharmaceutical contamination of dietary supplements is an emerging concern. Studies in other regions, such as Wu et al., have shown similar patterns of pharmaceutical residues in various food products, including seafood- and plant-based supplements, highlighting the global nature of this issue [52]. The detection of pharmaceutical residues in algal supplements, even at low concentrations, warrants further investigation. With increasing pharmaceutical consumption and their persistence in sediments, the likelihood of bioaccumulation in primary producers such as algae is expected to rise. Although the concentrations observed in our study were low, environmental levels of pharmaceuticals have been shown to affect the metabolism of aquatic organisms [53]. Similar effects in algae cannot be ruled out. Such alterations may lead to changes in metabolic pathways, the formation of unknown metabolites, or a reduction in the nutritional quality of the biomass. These potential impacts on algal metabolism highlight the need for a better understanding of the ecological consequences of chronic, low-level pharmaceutical exposure in aquatic environments.

The present study demonstrated a broad range of heavy metal concentrations in commercially available dietary supplements based on *Chlorella* and *Spirulina*. Among the 17 elements detected, Al, Mn, Sr, and Zn were the most abundant in both product types. These findings suggest that microalgae-based supplements tend to accumulate specific trace elements, with variations likely reflecting differences in cultivation environments, water quality, processing methods, and potentially contamination from manufacturing equipment or packaging materials [54,55]. Tl, a highly toxic element even at trace levels, was detected in the lowest concentrations among all the detected metals. A comparable distribution of elements was found in *Spirulina*-based supplements, where Al, Mn, Sr, and Zn also appeared as the major constituents. The observed inter-product differences further underscore the lack of standardization in raw material sourcing and manufacturing processes. Although Cd was present at relatively low levels in *Spirulina* samples, its detection remains significant due to its cumulative toxicity and potential carcinogenicity, particularly when ingested over prolonged periods through multiple dietary sources [56,57].

When comparing the sorption mechanisms of *Spirulina platensis* and *Chlorella vulgaris*, *Chlorella* is generally regarded as a more effective biosorbent under various conditions due to its higher equilibrium sorption capacity [58]. However, in our study, no clear predominance of *Chlorella* over *Spirulina* in metal content was observed. Specifically, higher levels of Mn and Rb were found in *Chlorella*, while *Spirulina* showed greater accumulation of Al, Cu, Ni, and Pb. This variation suggests that metal content is influenced not only by species-specific uptake mechanisms but also by external factors such as cultivation conditions. Bioaccumulation abilities have been reported for *Chlamydomonas reinhardtii*, which biosorbs multiple metals (Cu, Mn, As, Ni, Zn, Cd, U) and xenobiotics such as *o*-nitrophenol and carbamazepine. Its tolerance to heavy metals is linked to metallothionein gene expression [59]. Importantly, none of the analyzed samples from either group exceeded the maximum permissible concentrations for toxic metals such as Pb and Cd as specified by the European Commission regulations (e.g., EC No. 488/2014 for Cd: 0.050 µg/g; EC No. 2015/1005 for Pb: 0.30 µg/g) [13]. These results align with previous findings reporting detectable, but compliant, levels of heavy metals in both plant-based products and aquatic organisms [58]. However, regulatory compliance does not necessarily guarantee the absence of health risks. The bioaccumulative nature of certain metals and their chronic exposure potential highlight the need for health risk assessments, including EDI and contribution percentages to the guideline values—TWI (%), and to evaluate safety more comprehensively [58]. Despite these relatively high contributions, it is important to emphasize that, under the recommended dosage regimens, none of the examined supplements exceeded the established safety thresholds for TWI or other relevant toxicological benchmarks.

Nevertheless, considering their potential contribution to total exposure, especially when combined with other dietary sources, regular monitoring of elemental contents in microalgae supplements remains essential. This is particularly relevant for Al, a non-essential element with known neurotoxic potential. Its accumulation in the human body—especially in the brain, bones, and kidneys—has raised concern due to possible associations with neurodegenerative diseases such as Alzheimer’s disease [60]. In our study, several supplements—both *Chlorella* and *Spirulina*—contributed up to 56% of the TWI for Al established by EFSA (2 µg/g bw/week) [26]. In the general diet, aluminum exposure mainly comes from cereals, vegetables, tea, food additives, and leaching from cookware and packaging [61,62]. Algae-based supplements with elevated Al levels can significantly increase total intake, especially when combined with other sources. Regular consumption may thus pose health risks, particularly for sensitive groups such as children, elderly people, or individuals with impaired kidney function [63].

The comparison of elemental content between organically and conventionally cultivated microalgae-based supplements revealed no statistically significant differences for the majority of detected elements, including Al, Mn, Sr, and Zn. This finding suggests that, in general, producer-declared cultivation system (organic or conventional) does not exert a decisive influence on the elemental composition of the final product. These results are consistent with previous studies, which have similarly reported minor variations in trace metal concentrations between organic and conventional microalgae products [64]. However, further analyses on a larger group of supplements are needed to confirm these observations.

An exception was noted for V in *Spirulina*-based supplements, where significantly higher levels were observed in products derived from conventional cultivation. This difference may reflect variations in fertilizers, water sources, or nutrient formulations applied during production [65]. Although vanadium is not currently regulated in dietary supplements, its dual nature—as both a potentially beneficial trace element and a toxicant at elevated exposures—warrants close monitoring. Given that some consumers ingest such products daily, even moderate elevations in V concentration may be relevant from a toxicological standpoint [66]. Although organic labeling is often perceived as a marker of lower contamination risk, our data indicate that such an assumption may not hold uniformly true for all elements. While lower levels of vanadium in organic *Spirulina* are noteworthy, other elemental concentrations did not differ significantly between producer-declared cultivation systems. It should also be noted that microalgae are often cultivated in open pond systems, where environmental microorganisms may contribute to the overall contaminant profile. This highlights the need for further investigation into the correlation between organic certification protocols and actual contaminant profiles. Additional regulatory measures may be necessary to ensure consistent safety and quality of microalgae-based dietary supplements.

Our analysis of *Spirulina*- and *Chlorella*-based dietary supplements revealed distinct contamination profiles, characterized by both pharmaceutical residues and heavy metals. Partial Least Squares Discriminant Analysis identified thiabendazole, caffeine, and carbamazepine as key discriminators, while nickel emerged as the only heavy metal with statistically significant classification relevance. The detection of pharmaceuticals associated with wastewater effluents suggests environmental contamination, especially given the vulnerability of open or semi-controlled cultivation systems. Nickel concentrations observed in our samples align with those reported in the literature, reinforcing its analytical significance. Though other metals such as lead, cadmium, and arsenic were present at low levels, chronic exposure risks, particularly in sensitive populations, warrant concern.

It is worth emphasizing that the present study was based on a limited number of commercially available products purchased from reliable sources, and therefore the results may not fully represent the quality of all algal supplements available on the market. The results underscore the need for harmonized regulations, transparent labeling, and standardized monitoring protocols to ensure product safety and integrity in the expanding global market for microalgae-based supplements.

## 4. Materials and Methods

### 4.1. Sample Collection and Chemicals

The materials analyzed in this study comprised *Spirulina platensis* and *Chlorella vulgaris* dietary supplements, commercially available in tablet and powder forms. In total, 29 *Spirulina* and 23 *Chlorella* products were obtained from specialized online retailers. The samples represented a random selection of the most popular microalgae-based supplements available on the Polish market, differing in brand, country of origin, and certification status (organic or conventional). All products were purchased from reliable sources, including pharmacies and certified health food stores, to ensure market representativeness. Detailed characteristics of the analyzed supplements are provided in Table A6.

Pharmaceutical standards used in this study were of high-purity grade (>98%) and were purchased from Sigma-Aldrich (Darmstadt, Germany), Toronto Research Chemicals (Vaughan, ON, Canada), or obtained from the Drug Research Institute in Warsaw, Poland. Solvents, including acetonitrile (hypergrade for LC-MS/MS, LiChrosolv) and formic acid (98%), were obtained from Merck (Darmstadt, Germany). Ultrapure water was prepared using a Millipore system (Milli-Q water, Merck, Darmstadt, Germany).

Individual standard stock solutions of pharmaceuticals were prepared by dissolving the compounds in methanol at concentrations of 0.5 mg·mL^−1^ or 1 mg·mL^−1^, and stored at −20 °C for use in the preparation of working solutions. Isotope-labeled internal standards (^13^C, D) were employed to ensure accurate quantification: acetaminophen D4, atorvastatin D5, bisoprolol D5, clindamycin D3, erythromycin C13 D3, fluoxetine D5, metformin D6, mycophenolic acid D3, salbutamol D7, sulfamethoxazole D4, trimethoprim D9, valsartan D3.

A total of 134 pharmaceutical compounds were included in the analysis, categorized according to the Anatomical Therapeutic Chemical (ATC) classification system. Pharmaceuticals were selected based on our previous studies detecting these compounds in surface waters.

The compounds were distributed across the following categories: nervous system (43), cardiovascular system (26), anti-infectives for systemic use (35), alimentary tract and metabolism (7), respiratory system (7), genito-urinary system and sex hormones (4), antiparasitic products, insecticides and repellents (6), musculo-skeletal system (2), antineoplastic and immunomodulating agents (1, mycophenolic acid), and ecosystem-related substances (2, microcystins). 1-Naphthoxyacetic acid may also originate from anthropogenic sources, as it is a known metabolite of propranolol, a β-blocker drug.

### 4.2. Sample Preparation

#### 4.2.1. Metal Analysis

For the determination of elemental composition, approximately 200 mg of each sample, as well as the certified reference material, was accurately weighed using an analytical balance. Samples were transferred to Teflon PFA digestion vessels and subjected to acid digestion with a mixture of 4 mL of 65% HNO_3_ and 30% H_2_O_2_ (Suprapur^®^, Merck, Darmstadt, Germany) in a 3:1 (*v*/*v*) ratio. Microwave-assisted digestion was performed using a Multiwave 3000 system (Anton Paar, Ashland, VA, USA) at 200 °C for 15 min. The system automatically regulated power to maintain optimal digestion parameters, with operational limits of 300 °C and 150 bar. After digestion, samples were cooled to room temperature, quantitatively transferred to volumetric flasks, and diluted to 25 mL with high-purity deionized water (Direct-Q UV, Millipore, resistivity ~18.0 MΩ·cm). Metal concentrations were determined using inductively coupled plasma-based methods. Calibration was performed using multi-element standard solutions (ICP Multi-Element Standard Solution, Fluka Analytical, Saint Louis, MO, USA) prepared at various concentration levels. Method accuracy and precision were verified through a recovery study using Polish Certified Reference Material (Herbapol S.A., Lublin, Poland). The recovery rates exceeded 96%, confirming method reliability under reproducible analytical conditions.

#### 4.2.2. Pharmaceutical Analysis

For pharmaceutical residue analysis, 750 mg of powdered *Spirulina* or *Chlorella* was suspended in 1200 µL of acetonitrile containing formic acid, 10% aqueous sodium edetate, and internal standard solution (100 ng·mL^−1^), in a volumetric ratio of 1000:1:10:10. The suspension was vigorously shaken for 10 min, followed by centrifugation at 6720× *g* for 5 min. The resulting supernatant was transferred to a 2 mL Eppendorf tube, mixed with 300 µL of ultrapure water, adjusted to 1.5 mL with acetonitrile, and supplemented with 300 mg of ammonium acetate. After 5 min of shaking and centrifugation (6720× *g*, 5 min), the upper layer was transferred to a tube containing 100 mg of C18 octadecyl-endcapped sorbent, shaken for 3 min, and centrifuged again under the same conditions. The clarified extract was evaporated at 40 °C to dryness, reconstituted in 20 µL of methanol/water (1:1, *v*/*v*), followed by the addition of 80 µL of water. The final solution was centrifuged at 9300× *g* for 10 min, and the supernatant was subjected to LC-MS/MS analysis.

### 4.3. Analysis

Metal concentrations in the samples were quantified using Inductively Coupled Plasma Mass Spectrometry (ICP-MS) with instrumentation from Thermo Electron Corp (City). Calibration was performed using a commercially available multi-element standard solution (Fluka Analytical, MO 63103, USA), which was systematically diluted in 1% HNO_3_ to generate calibration curves spanning a concentration range of 1 ng/mL to 1000 ng/mL. PhACs concentrations were analyzed by high-performance liquid chromatography coupled to mass spectrometry with a Hybrid Triple Quadrupole/Linear Ion trap mass spectrometer (QTRAP^®^4000, AB SCIEX, Framingham, MA, USA). LC analysis was performed using an Agilent 1260 Infinity (Agilent Technologies, Santa Clara, CA, USA) equipped with a degasser, thermostated autosampler, and binary pump, and connected in series to an AB SCIEX 4000 QTRAP mass spectrometer equipped with a Turbo Ion Spray source that was operated in both positive mode and negative mode. The target compounds were analyzed in multiple reaction monitoring (MRM) as described previously [18]. The MRM transitions are presented in Table A7. The LC-MS analysis was performed using Agilent 1260 G1312B pump. The column was Kinetex 2.6 µm C 18 100 Å 100 × 4.6 mm (Phenomenex, Torrance, CA, USA). The oven temperature was 40 °C. The mobile phases were: A: H_2_O:HCOOH 998:2 (*v*/*v*), B: acetonitryle:HCOOH 998:2 (*v*/*v*). The samples were injected at volume of 10 μL. The flow rate was 500 μL∙min^−1^. The chromatographic separation was performed using the following gradient program: the initial composition was 80% phase A and 20% phase B, held for 1 min. The proportion of phase B was then increased linearly to 95% over 2 min and maintained for 6 min.

### 4.4. Calculations and Statistical Analysis

All measurements were conducted in three replicates. The obtained results were analyzed using Statistica software (version 10, StatSoft, TIBCO Software, Warsaw, Poland). Data were reported as mean values with standard deviation; however, statistical significance was assessed based on the median, upper, and lower quartiles. Due to the non-normal distribution and heterogeneity of variance in the dataset, nonparametric tests were applied. The Mann–Whitney U test was used for comparing two independent groups, with statistical significance set at *p* < 0.05. The statistical analysis based on the summary content of heavy metals and pharmaceuticals were performed as principal component analysis (PCA), partial least squares—discriminant analysis (PLS-DA), cross-validation for model (CV) and variable importance in projection (VIP) using MetaboAnalyst^®^ 6.0 online software.

Detection percentage (Det%) for the pharmaceutical was calculated as ratio:

$$Det\%=\frac{n}{n_{G}}$$

where

n—number of detections of a specific pharmaceutical
n_G_—total number of pharmaceutical detections (i.e., all pharmaceuticals detected)

Due to the unequal number of pharmaceuticals present in each therapeutic group, a relative detection frequency coefficient (${coeff}_{rel})$ was introduced to allow for meaningful comparison between groups. This normalization accounts for differences in group size and ensures that detection frequency is not biased by the absolute number of compounds in each category.

$${coeff}_{rel}=\frac{n_{Gdt}}{N_{G}}$$

where

n_Gdt_—total number of detections for all samples of compounds from compound group “G”.
N_G_—number of compounds from compound group “G”. Group G is the therapeutic class or pharmacological category of drugs in this analysis.

The estimated weekly intake (EWI) of selected trace elements and heavy metals was determined based on the dosage recommendations provided by different manufacturers, which ranged from 2–3 g up to 10 g per day, with a maximum daily intake limit of 15 g. Calculations were performed assuming an average body weight of 70.0 kg. The tolerable weekly intake (TWI) values were considered relative to body weight, with an assumed average adult weight of 70.0 kg for these calculations.

The EWI for each metal was calculated using the following equation:

$$EWI\;(\frac{mg}{week})=\frac{weekly\;consumption\;(mg/week)\times metal\;concentration\;(µg/g)}{average\;body\;weigh}$$

## 5. Conclusions

Both *Spirulina* and *Chlorella* preparations were found to contain not only trace metals but also residues of pharmaceutical compounds. Particular attention should be given to the presence of aluminum, which may contribute substantially to the tolerable weekly intake, as well as to sulfonamides, whose accumulation—especially with high consumption of algal supplements—could promote the selection of antimicrobial resistance. Notably, this is the first study to report the presence of pharmaceutical residues in commercially available algal-based supplements. These findings underscore the need for systematic monitoring of such products, particularly in light of the growing environmental burden of pharmaceutical contamination.

## Figures and Tables

**Table 1:** Estimated weekly intake (EWI) of each detected toxic metal and contribution percentages to the guideline values—TWI (%).

No.	EWI (mg/week)			EWI (µg/week)				TWI (%)						
	Al	Mn	Zn	Cd	Cr	Ni	Pb	Al	Mn	Zn	Cd	Cr	Ni	Pb
1	0.020	0.029	0.015	0.001	0.25	0.001	0.100	1.00	0.04	0.003	0.0002	0.0155	0.00004	0.006
2	0.501	0.070	0.021	0.180	3.38	0.069	1.965	25.04	0.10	0.004	0.0367	0.2069	0.00282	0.112
3	0.020	0.006	0.003	0.001	0.28	0.001	0.100	1.00	0.01	0.001	0.0002	0.0172	0.00004	0.006
4	0.015	0.024	0.003	0.012	0.25	0.005	0.075	0.75	0.03	0.001	0.0025	0.0151	0.00020	0.004
5	0.075	0.057	0.016	0.005	0.60	0.004	0.375	3.73	0.08	0.003	0.0009	0.0368	0.00016	0.021
6	0.015	0.020	0.007	0.014	0.26	0.007	0.075	0.75	0.03	0.001	0.0029	0.0159	0.00028	0.004
7	0.030	0.038	0.009	0.010	0.23	0.002	0.150	1.49	0.06	0.002	0.0021	0.0143	0.00006	0.009
8	0.020	0.024	0.006	0.001	0.54	0.023	0.100	1.00	0.04	0.001	0.0002	0.0334	0.00094	0.006
9	0.103	0.026	0.010	0.001	0.24	0.001	0.100	5.13	0.04	0.002	0.0002	0.0150	0.00004	0.006
10	0.023	0.032	0.012	0.001	0.73	0.017	0.115	1.15	0.05	0.002	0.0003	0.0448	0.00069	0.007
11	0.142	0.042	0.007	0.002	0.03	0.001	0.125	7.12	0.06	0.001	0.0003	0.0017	0.00005	0.007
12	0.020	0.024	0.005	0.001	0.02	0.001	0.100	1.00	0.03	0.001	0.0002	0.0013	0.00004	0.006
13	0.119	0.021	0.005	0.001	0.02	0.001	0.100	5.93	0.03	0.001	0.0002	0.0013	0.00004	0.006
14	0.020	0.019	0.009	0.001	0.02	0.006	0.100	1.00	0.03	0.002	0.0002	0.0013	0.00023	0.006
15	0.050	0.052	0.016	0.003	0.05	0.003	0.250	2.49	0.08	0.003	0.0006	0.0033	0.00010	0.014
16	0.578	0.077	0.030	0.120	0.08	0.004	0.375	28.92	0.11	0.006	0.0245	0.0050	0.00016	0.021
17	0.015	0.018	0.006	0.001	0.02	0.001	0.075	0.75	0.03	0.001	0.0002	0.0010	0.00003	0.004
18	0.059	0.005	0.002	0.015	0.01	0.004	0.025	2.94	0.01	n.d.	0.0031	0.0003	0.00015	0.001
19	0.020	0.021	0.010	0.001	0.02	0.001	0.100	1.00	0.03	0.002	0.0002	0.0013	0.00004	0.006
20	0.008	0.001	0.006	n.d.	0.01	n.d.	0.039	0.39	n.d.	0.001	0.0001	0.0005	0.00002	0.002
21	0.015	0.015	0.005	0.001	0.02	0.001	0.075	0.75	0.02	0.001	0.0002	0.0010	0.00003	0.004
22	0.075	0.134	0.022	0.005	0.08	0.004	0.375	3.73	0.19	0.005	0.0009	0.0050	0.00016	0.021
23	0.343	0.036	0.013	0.002	0.04	0.002	0.188	17.13	0.05	0.003	0.0005	0.0025	0.00008	0.011
24	0.085	0.035	0.005	0.0390	0.87	0.001	0.075	4.27	0.05	0.001	0.0080	0.0532	0.00003	0.004
25	0.075	0.017	0.009	0.0045	0.72	0.004	0.375	3.73	0.02	0.002	0.0009	0.0441	0.00016	0.021
26	0.049	0.004	0.001	0.0003	0.36	n.d.	0.025	2.47	0.01	n.d.	0.0001	0.0221	0.00001	0.001
27	0.005	0.003	0.001	0.0003	0.25	0.009	0.025	0.25	n.d.	n.d.	0.0001	0.0151	0.00036	0.001
28	0.050	0.016	0.031	0.0030	0.05	0.117	0.279	2.49	0.02	0.006	0.0006	0.0033	0.00477	0.016
29	0.123	0.019	0.006	0.0240	1.35	0.044	0.405	6.14	0.03	0.001	0.0049	0.0826	0.00178	0.023
30	0.050	0.007	0.006	0.0150	0.34	0.026	0.333	2.51	0.01	0.001	0.0031	0.0207	0.00107	0.019
31	0.104	0.007	0.004	0.0009	0.66	0.001	0.075	5.22	0.01	0.001	0.0002	0.0406	0.00003	0.004
32	0.030	0.074	0.015	0.1440	0.91	0.028	0.150	1.49	0.11	0.003	0.0294	0.0555	0.00113	0.009
33	0.010	0.006	0.003	0.0006	0.01	0.001	0.050	0.50	0.01	0.001	0.0001	0.0007	0.00002	0.003
34	0.010	0.007	0.002	0.0220	0.20	0.003	0.050	0.50	0.01	n.d.	0.0045	0.0123	0.00013	0.003
35	n.d.	n.d.	n.d.	n.d.	0.81	0.001	0.969	8.41	0.03	0.001	0.0061	0.0498	0.00003	0.055
36	0.012	0.006	0.003	0.0008	0.67	0.006	0.340	0.62	0.01	0.001	0.0002	0.0409	0.00023	0.019
37	0.025	0.012	0.007	0.1350	0.03	0.029	0.125	1.24	0.02	0.001	0.0276	0.0017	0.00118	0.007
38	0.133	0.013	0.009	0.0012	0.02	0.001	0.100	6.63	0.02	0.002	0.0002	0.0013	0.00004	0.006
39	1.122	0.040	0.020	0.0030	0.05	0.037	0.250	56.10	0.06	0.004	0.0006	0.0033	0.00150	0.014
40	0.369	0.018	0.010	0.0550	0.03	0.001	0.125	18.44	0.03	0.002	0.0112	0.0017	0.00005	0.007
41	0.075	0.041	0.018	0.1800	0.08	0.004	0.375	3.73	0.06	0.004	0.0367	0.0050	0.00016	0.021
42	0.158	n.d.	0.005	0.0018	0.03	0.009	0.150	7.90	n.d.	0.001	0.0004	0.0020	0.00035	0.009
43	0.055	n.d.	0.002	0.0085	0.01	n.d.	0.027	2.74	n.d.	n.d.	0.0017	0.0004	0.00001	0.002
44	0.020	n.d.	0.004	0.0012	0.02	0.001	0.100	1.00	n.d.	0.001	0.0002	0.0013	0.00004	0.006
45	0.015	n.d.	0.003	0.0009	0.02	0.001	0.075	0.75	n.d.	0.001	0.0002	0.0010	0.00003	0.004
46	0.184	n.d.	0.006	0.0270	0.02	0.011	0.075	9.19	n.d.	0.001	0.0055	0.0010	0.00045	0.004
47	0.120	n.d.	0.008	0.0009	0.02	0.001	0.075	6.00	n.d.	0.002	0.0002	0.0010	0.00003	0.004
48	0.075	n.d.	0.015	0.3600	0.08	0.004	0.375	3.73	n.d.	0.003	0.0735	0.0050	0.00016	0.021
49	0.451	n.d.	0.050	0.0045	0.08	0.004	0.375	22.57	n.d.	0.010	0.0009	0.0050	0.00016	0.021
50	0.037	n.d.	0.017	0.0023	0.04	0.002	0.188	1.87	n.d.	0.003	0.0005	0.0025	0.00008	0.011
51	0.005	0.002	0.001	0.0003	0.39	0.003	0.025	0.25	n.d.	n.d.	0.0001	0.0236	0.00013	0.001
52	0.400	0.139	0.028	0.0030	0.05	0.060	0.250	20.01	0.20	0.006	0.0006	0.0033	0.00244	0.014

n.d.—not detected.

**Table 2:** Maximal concentration (c_max_), mean concentration (c_mean_), concentration median and concentration range for the detected pharmaceuticals among all tested samples.

Compound	c_max_ [ng/g]	c_mean_ [ng/g]	Median	Range
Atenolol	2.59	0.07	0.02	2.57
Atorvastatin	7.67	1.63	1.37	6.30
Atropine	0.97	0.07	0.05	0.92
Benzocaine	38.27	3.18	0.32	37.95
Caffeine	27.93	1.80	1.34	27.91
Carbamazepine	1.33	0.11	0.02	1.31
Clindamycin	0.20	0.04	0.04	0.16
Enalapril	0.39	0.02	0.01	0.38
Erythromycin	0.47	0.07	0.04	0.43
Fleroxacin	1.38	0.18	0.04	1.34
Guaifenesin	1.24	0.06	0.01	1.23
Imipramine	0.39	0.06	0.05	0.34
Lincomycin	0.38	0.01	0.01	0.38
Mebendazole	1.01	0.10	0.02	0.99
Melatonin	0.24	0.02	0.02	0.22
Metronidazole	0.90	0.19	0.01	0.90
Mirtazapine	0.53	0.04	0.03	0.50
Paroxetine	8.36	0.47	0.31	8.05
Promazine	0.71	0.09	0.07	0.63
Sildenafil	6.90	1.18	0.20	6.70
Sotalol	0.66	0.03	0.02	0.64
Sulfamethoxazole	1.56	0.36	0.06	1.50
Sulfathiazole	48.83	1.12	0.07	48.76
Thiabendazole	0.82	0.36	0.52	0.81
Tiapride	0.31	0.02	0.01	0.30
Tolperison	0.43	0.02	0.01	0.41
Trimethoprim	0.42	0.08	0.01	0.40
Tramadol	0.04	0.01	0.01	0.04
Zolpidem	0.21	0.02	0.02	0.19

## Data Availability

The original contributions presented in this study are included in the article/Appendix A. Further inquiries can be directed to the corresponding author(s).

## Abbreviations

The following abbreviations are used in this manuscript:

ATC	Anatomical Therapeutic Chemical (classification system)
bw	body weight
CV	cross-validation (for model)
Det%	detection percentage
EC	European Commission
EFSA	European Food Safety Authority
EWI	estimated weekly intake
H_2_O_2_	hydrogen peroxide
HNO_3_	nitric acid
ICP	inductively coupled plasma
ICP-MS	inductively coupled plasma mass spectrometry
LC	liquid chromatography
LC-MS	liquid chromatography–mass spectrometry
LC-MS/MS	liquid chromatography–tandem mass spectrometry
MIC	minimum inhibitory concentration
MRM	multiple reaction monitoring
PCA	principal component analysis
PhACs	pharmaceutically active compounds
PLS-DA	partial least squares–discriminant analysis
PTWI	provisional tolerable weekly intake
QTRAP^®^	hybrid triple quadrupole/linear ion trap mass spectrometer
TWI	tolerable weekly intake
VIP	variable importance in projection
TWI (%)	percentage of the tolerable weekly intake
USA	United States of America

## Appendix A

**Table A1:** The levels of heavy metals and trace elements [µg/g] in the tested samples of *Chlorella* (*n* = 23) and *Spirulina* (*n* = 29) supplements.

	*Chlorella*			*Spirulina*		
Elements	Mean ± SD	Min	Max	Mean ± SD	Min	Max
Al	148 ± 163	49.8	588	269 ± 267	49.7	1122
Ba	6.0 ± 3.3	0.23	10.1	7.5 ± 5.6	0.23	20.9
Cd	0.022 ± 0.041	0.003	0.150	0.059 ± 0.081	0.003	0.270
Co	0.52 ± 0.16	0.154	0.731	0.43 ± 0.15	0.162	0.687
Cr	0.45 ± 0.60	0.054	2.25	1.1 ± 1.4	0.054	4.49
Cs	0.025 ± 0.023	0.006	0.069	0.15 ± 0.20	0.006	0.693
Cu	4.0 ± 3.5	1.55	16.3	14 ± 31	0.80	104
Ga	0.193 ± 0.091	0.015	0.338	0.24 ± 0.16	0.015	0.603
Mn	75 ± 31	6.6	110	47 ± 37	11.3	139
Mo	0.19 ± 0.07	0.112	0.287	0.37 ± 0.68	0.089	2.59
Ni	0.8 ± 1.7	0.09	8.28	16 ± 35	0.47	106
Pb	0.30 ± 0.22	0.25	1.31	0.65 ± 0.85	0.25	3.23
Rb	3.5 ± 1.6	0.15	6.65	2.5 ± 1.0	0.88	4.74
Sr	16 ± 16	5.5	60.9	18.0 ± 9.8	7.0	43.6
Tl	0.015 ± 0.015	0.002	0.049	0.07 ± 0.11	0.02	0.43
V	10 ± 32	0.15	101	0.46 ± 0.20	0.20	0.73
Zn	18.9 ± 8.2	7.1	41.2	18.1 ± 8.7	5.4	42.7

**Table A2:** Elements content in *Chlorella* supplements depending on the form of the cultivation method.

	Conventional Growing *n* = 9		Organic Growing *n* = 14		
Elements	Mean	SD	Mean	SD	*p* Value
Elements Content [µg/g]					
Al	177.3	198.6	132.0	146.5	0.699
Ba	7.30	2.33	5.40	3.70	0.494
Cd	0.023	0.052	0.021	0.036	0.974
Co	0.601	0.136	0.480	0.167	0.362
Cr	0.451	0.645	0.447	0.593	0.821
Cs	0.017	0.020	0.029	0.024	0.649
Cu	3.33	2.15	4.34	4.07	0.561
Ga	0.209	0.048	0.186	0.107	0.649
Mn	82.7	24.3	71.6	34.7	0.872
Mo	0.174	0.098	0.193	0.055	0.494
Ni	1.51	2.83	0.368	0.536	0.519
Pb	0.250	0.000	0.321	0.274	0.821
Rb	4.75	1.66	2.99	1.35	0.138
Sr	9.72	1.19	18.3	19.3	0.649
Tl	0.0227	0.0078	0.0118	0.0171	0.111
V	0.366	0.317	0.359	0.240	1.000
Zn	17.7	5.28	19.5	9.58	0.872

**Table A3:** Elements content in *Spirulina* supplements depending on the form of the cultivation method.

	Conventional Growing *n* = 18		Organic Growing *n* = 11		
Elements	Mean	SD	Mean	SD	*p* Value
Elements Content [µg/g]					
Al	216.25	186.13	334.5	339.42	0.469
Ba	9.48	5.84	4.37	3.73	0.164
Cd	0.039	0.049	0.085	0.105	0.357
Co	0.474	0.142	0.358	0.146	0.213
Cr	1.38	1.55	0.69	1.22	0.324
Cs	0.093	0.066	0.235	0.306	0.942
Cu	10.18	25.28	19.09	36.92	0.511
Ga	0.291	0.173	0.165	0.125	0.188
Mn	36.49	14.5	60.3	50.78	0.494
Mo	0.196	0.152	0.646	1.088	0.421
Ni	8.51	26.02	25.32	44.12	0.584
Pb	0.97	1.06	0.25	0.01	0.267
Rb	2.89	1.06	1.95	0.67	0.124
Sr	18.54	12.05	17.24	5.89	0.826
Tl	0.087	0.137	0.04	0.026	0.38
V	0.56	0.18	0.29	0.1	* 0.023
Zn	16.72	9.68	19.7	7.49	0.167

* *p* < 0.05 indicates statistically significant differences between groups.

**Table A4:** Provisional tolerable weekly intake (PTWI) values for trace elements and toxic metals.

Element	PTWI (μg/week/kg bw)	Reference	PTWI (μg/week/70 kg bw)
Al	28.6	FAO/WHO [67]	2000
Cd	7	FAO/WHO [68]	490
Cr	23.3	FAO/WHO [68]	1631
Cu	3500	FAO/WHO [68]	245,000
Fe	5600	FAO/WHO [68]	392,000
Mn	980	EPA (2014) [69]	68,600
Ni	35	FAO/WHO [68]	2450
Pb	25	FAO/WHO [68]	1750
Zn	7000	FAO/WHO [68]	490,000

**Table A5:** Maximal concentration (c_max_), mean concentration (c_mean_), concentration median and concentration range for the detected pharmaceuticals among *Chlorella-* and *Spirulina*-based tested samples.

Compound	*Chlorella*				*Spirulina*			
	c_max_ [ng/g]	c_mean_ [ng/g]	Median	Range	c_max_ [ng/g]	c_mean_ [ng/g]	Median	Range
Atenolol	0.02	0.02	0.02	0	2.59	0.12	0.02	2.57
Atorvastatin	7.67	1.90	1.37	6.30	1.37	1.37	1.37	0
Atropine	0.97	0.09	0.05	0.92	0.05	0.05	0.05	0
Benzocaine	38.27	5.76	0.32	37.95	10.01	0.71	0.32	9.69
Caffeine	4.50	1.91	1.86	4.47	27.93	1.70	0.19	27.91
Carbamazepine	1.33	0.19	0.06	1.31	0.3	0.03	0.02	0.28
Clindamycin	0.20	0.04	0.04	0.16	0.04	0.04	0.04	0
Enalapril	0.01	0.01	0.01	0	0.39	0.03	0.01	0.38
Erythromycin	0.47	0.06	0.04	0.43	0.37	0.09	0.04	0.33
Fleroxacin	0.04	0.04	0.04	0	1.38	0.31	0.04	1.34
Guaifenesin	0.21	0.06	0.01	0.20	1.24	0.06	0.01	1.23
Imipramine	0.05	0.05	0.05	0	0.39	0.07	0.05	0.34
Lincomycin	0.01	0.01	0.01	0	0.38	0.02	0.01	0.38
Mebendazole	0.92	0.07	0.02	0.90	1.01	0.13	0.02	0.99
Melatonin	0.02	0.02	0.02	0	0.24	0.03	0.02	0.22
Metronidazole	0.90	0.11	0.01	0.90	0.39	0.27	0.36	0.38
Mirtazapine	0.53	0.05	0.03	0.50	0.03	0.03	0.03	0
Paroxetine	0.31	0.31	0.31	0	8.36	0.63	0.31	8.05
Promazine	0.07	0.07	0.07	0	0.71	0.10	0.07	0.63
Sildenafil	6.90	1.61	0.20	6.70	3.42	0.78	0.20	3.22
Sotalol	0.02	0.02	0.02	0	0.66	0.05	0.02	0.64
Sulfamethoxazole	1.56	0.35	0.06	1.50	1.31	0.38	0.06	1.25
Sulfathiazole	0.76	0.17	0.07	0.69	48.83	2.03	0.07	48.76
Thiabendazole	0.82	0.19	0.01	0.81	0.59	0.51	0.53	0.58
Tiapride	0.01	0.01	0.01	0	0.31	0.03	0.01	0.3
Tolperison	0.21	0.02	0.01	0.20	0.43	0.03	0.01	0.41
Trimethoprim	0.42	0.07	0.01	0.40	0.40	0.08	0.01	0.39
Tramadol	0.04	0.02	0.01	0.04	0.03	0.01	0.01	0.03
Zolpidem	0.02	0.02	0.02	0	0.21	0.03	0.02	0.19

**Table A6:** Main information of tested samples of *Spirulina* and *Chlorella* supplements.

Code Number	Type of Supplement	Cultivation Method	Product Type	Manufacturing Company	Country of Origin
1	*C* *hlorella*	organic	powder	SuperFoods	China
2	*C* *hlorella*	organic	powder	Diet Food	Poland
3	*C* *hlorella*	organic	tablets	Diet Food	Poland
4	*C* *hlorella*	organic	powder	Natubio	Portugal
5	*C* *hlorella*	organic	powder	Bio Planet	South Korea
6	*C* *hlorella*	organic	tablets	Medicura	China
7	*C* *hlorella*	conventional	tablets	BOF	Poland
8	*C* *hlorella*	conventional	powder	Ekogram-Zielonki	Poland
9	*C* *hlorella*	organic	powder	Zielony smak	China
10	*C* *hlorella*	conventional	tablets	Hanoju	Korea
11	*C* *hlorella*	conventional	powder	Rainforest Foods	Denmark
12	*C* *hlorella*	organic	tablets	NaturaLine	China
13	*C* *hlorella*	conventional	tablets	Natvita	China
14	*C* *hlorella*	organic	powder	Alpha Superfoods	China
15	*C* *hlorella*	organic	powder	Naturya	Poland
16	*C* *hlorella*	organic	powder	SuperFoods	China
17	*C* *hlorella*	organic	tablets	Meridian	China
18	*C* *hlorella*	conventional	tablets	Aura Glob Trade	Taiwan
19	*C* *hlorella*	conventional	powder	Hanoju	Poland
20	*C* *hlorella*	organic	tablets	Solgar	Denmark
21	*C* *hlorella*	conventional	tablets	Rainforest Foods	USA
22	*C* *hlorella*	organic	powder	NatVita	China
23	*C* *hlorella*	organic	tablets	NatVita	China
24	*S* *pirulina*	organic	powder	Bogutyn Młyn	China
25	*S* *pirulina*	organic	powder	Diet Food	Poland
26	*S* *pirulina*	organic	powder	Dragon superfoods	India
27	*S* *pirulina*	conventional	tablets	Aura Herbals	Poland
28	*S* *pirulina*	organic	tablets	Vegan Bio	India
29	*S* *pirulina*	conventional	tablets	Targroch	China
30	*S* *pirulina*	conventional	tablets	Targroch	China
31	*S* *pirulina*	conventional	tablets	Biotech USA	Hungary
32	*S* *pirulina*	organic	tablets	GSE	Germany
33	*S* *pirulina*	conventional	tablets	Swanson	Poland
34	*S* *pirulina*	conventional	powder	Eka Medica	Poland
35	*S* *pirulina*	conventional	tablets	SuperFoods	China
36	*S* *pirulina*	conventional	tablets	MyVita	Poland
37	*S* *pirulina*	organic	powder	Rainforest Foods	USA
38	*S* *pirulina*	conventional	tablets	NaturaLine	China
39	*S* *pirulina*	organic	powder	Naturya	China
40	*S* *pirulina*	organic	tablets	SuperFoods	China
41	*S* *pirulina*	conventional	powder	Supernutrients	China
42	*S* *pirulina*	conventional	tablets	Natura Vitals BV	The Netherlands
43	*S* *pirulina*	conventional	tablets	Herbapol	Poland
44	*S* *pirulina*	conventional	powder	Hanoju	Deutschland
45	*S* *pirulina*	conventional	tablets	Solgar	USA
46	*S* *pirulina*	organic	tablets	Rainforest Foods	USA
47	*S* *pirulina*	organic	tablets	Cyanotech	USA
48	*S* *pirulina*	organic	powder	NatVita	China
49	*S* *pirulina*	conventional	powder	NatVita	China
50	*S* *pirulina*	organic	tablets	NatVita	China
51	*S* *pirulina*	conventional	tablets	AZ medica	Poland
52	*S* *pirulina*	organic	tablets	Cyanotech	USA

**Table A7:** MRM transitions of compounds determined in LC-MS/MS analysis. Abbreviations: CE—collision energy; DP—declustering potential; CXP—collision cell exit potential; EP—entrance potential.

Q1 [Da]	Q3 [Da]	Compound	DP [V]	EP [V]	CE [V]	CXP [V]
267	145	Atenolol 1	86	10	39	12
267	190	Atenolol 2	86	10	27	10
559	440	Atorvastatin 1	91	10	33	12
559	250	Atorvastatin 2	91	10	63	14
564	445	Atorvastatin-D5 1	106	10	33	12
564	255	Atorvastatin-D5 2	106	10	61	14
290	124	Atropine 1	91	10	35	8
290	93	Atropine 2	91	10	45	6
166	138	Benzocaine 1	56	10	17	10
166	120	Benzocaine 2	56	10	27	8
326	116	Bisoprolol 1	91	10	27	8
326	222	Bisoprolol 2	91	10	21	12
331	121	Bisoprolol-D5 1	96	10	29	8
331	89	Bisoprolol-D5 2	96	10	73	6
556	202	Caffeine 1	101	10	47	16
556	106	Caffeine 2	101	10	99	6
204	144	Caffeine-D9 1	56	10	31	12
204	116	Caffeine-D9 1	56	10	35	8
237	194	Carbamazepine 1	86	10	29	12
237	179	Carbamazepine 2	86	10	51	14
748	158	Clarithromycin 1	111	10	41	12
748	590	Clarithromycin 2	111	10	29	16
425	126	Clindamycin 1	91	10	43	8
425	377	Clindamycin 2	91	10	29	10
429	129	Clindamycin-D3 1	96	10	43	10
429	381	Clindamycin-D3 2	96	10	29	10
315	86	Clomipramine 1	81	10	29	6
377	234	Enalapril 1	76	10	29	4
377	117	Enalapril 2	76	10	55	8
734	158	Erythromycin 1	106	10	43	12
734	576	Erythromycin 2	106	10	29	14
738	162	Erythromycin-C13-D3 1	96	10	45	12
738	580	Erythromycin-C13-D3 2	96	10	29	16
370	326	Fleroxacin 1	81	10	29	8
370	269	Fleroxacin 2	81	10	39	6
199	125	Guaifenesin 1	66	10	15	10
199	163	Guaifenesin 2	66	10	13	10
281	86	Imipramine 1	76	10	25	6
281	58	Imipramine 2	76	10	59	10
362	318	Levofloxacin 1	86	10	29	8
362	261	Levofloxacin 2	86	10	41	16
407	126	Lincomycin 1	86	10	41	8
407	359	Lincomycin 2	86	10	27	10
296	264	Mebendazole 1	81	10	31	16
296	77	Mebendazole 2	81	10	71	14
233	174	Melatonin 1	61	10	19	12
233	159	Melatonin 2	61	10	37	12
172	128	Metronidazole 1	41	10	21	10
172	82	Metronidazole 2	41	10	37	14
266	195	Mirtazapine 1	46	10	37	10
266	72	Mirtazapine 2	46	10	33	4
362	318	Ofloxacin 1	96	10	29	18
362	261	Ofloxacin 2	96	10	41	16
330	70	Paroxetine 1	51	10	49	4
330	192	Paroxetine 2	51	10	31	14
285	86	Promazine 1	66	10	29	6
285	58	Promazine 2	66	10	57	10
315	176	Ranitidine 1	66	10	25	14
315	130	Ranitidine 2	66	10	35	8
824	791	Rifampicin 1	101	10	25	14
824	151	Rifampicin 1	101	10	47	14
475	283	Sildenafil 1	121	10	53	16
475	100	Sildenafil 2	121	10	43	6
273	255	Sotalol 1	61	10	19	16
273	213	Sotalol 2	61	10	27	18
251	156	Sulfadiazine 1	66	10	23	12
251	92	Sulfadiazine 2	66	10	39	6
254	156	Sulfamethoxazole 1	71	10	23	10
254	92	Sulfamethoxazole 2	71	10	41	6
258	160	Sulfamethoxazole-D4 1	76	10	25	12
258	96	Sulfamethoxazole-D4 2	76	10	43	6
256	156	Sulfathiazole 1	71	10	23	12
256	92	Sulfathiazole 2	71	10	41	6
202	131	Thiabendazole 1	96	10	47	8
202	175	Thiabendazole 2	96	10	37	12
329	256	Tiapride 1	71	10	29	14
329	213	Tiapride 2	71	10	45	16
246	98	Tolperison 1	51	10	25	6
246	70	Tolperison 2	51	10	55	12
291	230	Trimethoprim 1	96	10	33	14
291	261	Trimethoprim 2	96	10	35	16
300	234	Trimethoprim-D9 1	106	10	37	14
300	123	Trimethoprim-D9 2	106	10	35	8
264	58	Tramadol 1	51	10	45	10
264	42	Tramadol 2	51	10	125	4
308	235	Zolpidem 1	111	10	49	18
308	236	Zolpidem 2	111	10	37	18
